# Gender differences in dealing with recurrent implantation failure after fertility treatments: a foundation for adequate support models

**DOI:** 10.3389/frph.2025.1698380

**Published:** 2025-11-17

**Authors:** Pascale Stucki, Michèle Meier, Sarah Charlotte Maria di Giacopo, Kurt Birchler, Brigitte Leeners

**Affiliations:** 1 Department of Reproductive Endocrinology, University Hospital Zurich, Zurich, Switzerland; 2 Faculty of Medicine, University of Zurich, Zurich, Switzerland

**Keywords:** IVF/ICSI, recurrent implantation failure, psychosocial experience, gender differences, qualitative study

## Abstract

**Background:**

Understanding patients’ needs is mandatory to optimize medical support. Previous research has identified differences in male and female coping strategies. As embryo implantation failure poses a burden for both partners and as an intact partnership is beneficial to overcome infertility, support models should adjust to the needs of both partners. Psychosocial reactions to recurrent failure of fertility treatments were identified as one of the important but under-researched topics in reproductive medicine, and especially knowledge on gender-specific reactions to recurrent implantation failure (RIF) is sparse. We therefore explored male and female emotional/ psychosocial reactions and coping strategies in RIF.

**Methods:**

Women and men from heterosexual couples, where embryo transfers failed to result in pregnancy at least three consecutive times, participated in qualitative semi-structured interviews. Qualitative content analysis was conducted according to the content structuring model of Kuckartz and Rädiker.

**Results:**

Dyadic influence in conversation was apparent, with women being more verbally inclined with a vivid narrative style. Men showed a lower intensity and variety of emotional and psychosocial reactions with disappointment being dominant. Greater optimism, little negative impact on quality of life, and sexuality were key male findings. Feelings of guilt were significantly more common among women. Unexpectedly, gender differences in coping strategies were less pronounced, and shared strategies emerged.

**Discussion/conclusions:**

Experiences with RIF of affected women cannot be directly applied to male partners as men's emotional and psychosocial consequences differ in extent and quality. This gender gap should be considered to improve clinical practice.

## Introduction

Globally, 8%–12% of reproductive-aged couples experience infertility, often inducing a significant psychosocial burden (1). Recurrent implantation failure (RIF), defined as at least three consecutive transfers of good-quality embryos without any resulting pregnancy, increases such burden (2). As RIF occurs in approximately 10% of couples undergoing assisted reproductive technologies (ART) (3), a significant number face this situation.

Infertility and RIF as low-controlled, chronic stressors have added psychosocial dimensions, contrasting other health problems, where usually only one person in a couple is affected and the counterpart plays a purely supportive role. However, current literature on psychological consequences of unsuccessful fertility treatments is largely limited to anxiety, depression, and distress (4). Available studies therefore do not cover the full range of emotions seen in couples with RIF and completely lack the dyadic aspect.

Responses to stressors and utilized coping strategies vary between the genders (5). For example, women tend to react more in a depressive, anxious pattern when exposed to psychological stressors, which may reflect a greater vulnerability (6). However, other findings support comparable patterns in reactions to infertility in both genders, which differ in extent (5). As partners can be a valuable resource in dealing with the psychological burden associated with infertility (5), it is mandatory to better understand a couple's needs and strategies to cope with RIF as well as characteristics of dyadic aspects, as both partners individually face a shared problem.

In ART, psychological interventions can enhance well-being, reduce distress, and are associated with positive effects on pregnancy chances (4, 7, 8). In addition, psychological distress, alongside financial aspects, is the main reason for ART dropout. Tailored support systems may provide an added benefit in the reduction of psychological strain from RIF and therefore contribute to preventing premature ending of fertility treatment (9, 10).

While there are good practice recommendations (GPR) on causes and contributing factors in RIF (11), there are no universal guidelines for gender-specific psychosocial management and support for couples. The lack of gender-specific understanding of reactions to RIF further hampers adequate professional healthcare support systems. Care concepts that sufficiently meet men's needs facilitate their ability to provide emotional support, a core function of marriage (12). Current support efforts would therefore benefit from investigating gender-specific experiences as well as coping strategies in RIF, so that individual needs can be met, and a customized prophylactic psychological support system can be established.

The present evaluation aims to identify (i) qualitative and quantitative differences and similarities in emotional and psychosocial reactions, (ii) coping strategies between men and women experiencing RIF, and (iii) quantitative communication aspects and to (iv) analyze if eventual differences are rather gender or couple specific.

## Materials and methods

For this monocentric interview study, male and female partners within heterosexual couples with three consecutive single embryo implantation failures after fertility treatment were interviewed in 2023. No distinction was made between fresh or frozen cycles. The inclusion criteria included adequate proficiency in the German language, absence of any clinically significant condition potentially influencing study outcomes (current psychiatric diagnoses, non-infertility-related chronic illnesses), and written consent to study participation. The project was submitted to the cantonal ethics committee of Zurich, Switzerland, which decided that the project was in line with Swiss legislation and ethical regulations. The project was carried out in compliance with the Declaration of Helsinki. Adherence to ethical requirements was confirmed by the local ethics committee.

Couples followed the standardized clinical health and fertility investigations in preparation for a fertility treatment. In addition to the patients' history, laboratory parameters served to evaluate fertility and infection with hepatitis B and C and HIV. Men underwent semen analysis, and women underwent either hydrosonography, hydrocontrastsonography, or laparoscopy. Study participants were identified by revising medical charts of their preparation and realization of fertility treatments in the Department of Reproductive Endocrinology, University Hospital Zurich and invited to study participation via telephone or email. To allow for a more flexible schedule, reduce effort to participate, and address individual needs, interviews were offered on-site or online, as a joint or individual interview. Sample size was based on information saturation, i.e., the point at which all main categories related to the research question are fully developed and integrated (13). Consequently, couples were recruited and interviewed until no additional information could be obtained. Topics addressed were entered continuously into the data file after each interview, and recruitment was stopped when no new topics were mentioned. In total, 27 couples were contacted (overall response rate: 37.0%), and 10 decided to participate in the study. The main reasons for refusal were lack of time and fear of emotional intensification from the interview. Six couples took part in the interview together (two in person, four online) and four individually (three women and four men online, one woman in person). Altogether, 78.6% of the interviews were conducted online.

The qualitative methodology adopted a rather inductive approach, avoiding rigid predefined hypotheses (14). A guide for a semi-structured interview was designed based on a comprehensive review of existing literature (Di Giacopo et al., in review process) about the treatment-related emotional and psychosocial implications of RIF in men and women, supplemented by clinical expertise. *Emotional* refers to subjective feelings, whereas *psychosocial* encompasses an interplay between psychological and social effects, shaping behavioral and adaptive responses to a stressor (15). This resulted in 35 primary questions about key topics with optional follow-up questions. Examples of interview questions are shown below.

- Is there anything that has helped you, as a couple, manage the outcome of the negative pregnancy test, and has this changed during subsequent attempts to conceive?
- Have any aspects of your partnership changed during fertility treatment? If yes, please describe the nature of these changes.
- What differences have you observed between you and your partner in coping with repeated unsuccessful attempts to conceive?
- Were there any differences between you as a couple in the decision-making process? If so, please describe them.

Constructed with open-ended, narrative-generating questions and allowing for flexible sequencing, the guide prioritized spontaneous narratives, fostering a natural and empathetic interview atmosphere. Emerging topics were continuously integrated. Thus, it was ensured that preselected important topics were covered, while also allowing space for participants to introduce new topics.

Speech duration represents speaking time in minutes. Speech contribution is defined as the total speaking time as a proportion of the total interview duration, and a speech segment is considered a segment where the participant spoke during the interview.

The analytical process followed Rädiker and Kuckartz's (16) seven-step structured content analysis model. Initial phases facilitated by MAXQDA 2022 software (17) comprised transcription, coding of interview data according to predefined key topics and subcategories, and the creation of memos for annotation. Rigor and trustworthiness were ensured by several revision rounds of the transcripts as well as discussion of key topics and subcategories between the members of the research team. Subsequent stages included the identification of additional key topics, comprehensive coding of all data, and formulation of inductive subcategories. Percentages of study participants with findings matching a certain subcategory were calculated. For each participant, a speech segment count, the total speech duration in minutes, and speech contribution in percentage were determined based on the transcribed interviews. Overall relevant mean values and sample standard deviations were calculated using Microsoft Excel. Threshold percentages displayed in the manuscript were established based on clinical expertise. No statistical tests were conducted.

## Results

Table 1 summarizes sociodemographic data from the study group. Table 2 gives an overview of reactions and experiences in which the number of statements between men and women differed by ≥20%.

**Table 1:** Sociodemographic data from the study group.

	Characteristic	Women	Men
Total sample	*n*	10	10
Age (years)	Mean (SD) Range	38.0 (2.8) 33–43	39.0 (4.6) 33–49
Country of birth (*n*)	Switzerland	8	5
	Germany	1	3
	Bosnia and Herzegovina	0	1
	Greece	0	1
	Netherlands	1	0
Years living in CH	Mean (SD)	13.0 (7.1)	20.8 (10.7)
(2 ♀ and 5 ♂ born outside CH)	Range	8–18	10–38
Highest level of education (*n*)	Apprenticeship	2	0
	High school	0	1
	University	8	8
	Missing information	0	1
Duration of partnership (years)	Mean (SD)	9.0 (3.6)	
	Range	3–16	
Cause of infertility	Female factor Male factor Combined female/male factor Unexplained Desire for PID because of hereditary disease Male carrier Female carrier Both carrier	1 1 2 3 3 1 1 1 1	
Duration of infertility (years)	Mean (SD)	4.2 (2.0)	
	Range	2–6	
Duration since first evaluation of infertility (years)	Mean (SD)	3.4 (1.6)	
	Range	2–6	
Number of embryo transfers (ETs)	Mean (SD) of all ETs	5.5 (2.5)	
	Range of all ETs	3–11	
	Mean (SD) of ETs without pregnancy	4.9 (2.3)	
	Range of ETs without pregnancy	3–10	
Number of participants with already ≥1 child^a^	Spontaneous conception	2	3
	Through IVF/ICSI	2	2
Success of ART at the time of the interview (n)	Yes	4	4
Interval between last ET and interview (months)	Mean (SD)	7.0 (5.6)	7.0 (5.7)
	Range	2–16	1–16

SD, standard deviation; PID, preimplantation diagnosis.

a One man already had one child from a previous partnership. All other children were conceived in the current partnerships.

**Table 2:** Reactions to RIF with ≥20% differences in the number of statements between men and women.

Experiences	Specification	Women, *n* = 10	Men, *n* = 10	♀ > ♂	♂ > ♀
Emotional and psychological well-being	Guilt	7	1	60%	–
	Grief	8	3	50%	–
	Struggling with fate	6	1	50%	–
	Emotional rollercoaster^a^	4	1	30%	–
	Loneliness	3	1	20%	–
	Self-doubts^b^	2	–	20%	–
	Hopelessness^c^	3	1	20%	–
Quality of life (QOL)	Infertility as a major factor for reduced QOL	3	–	30%	–
	Infertility as a secondary factor related to QOL	2	–	20%	–
Self-perception	No influence on self-perception as a man/woman	4	7	–	30%
Social pressure	No perception of social pressure	2	4	–	20%
Social support	Desire for topic-related exchange with fellow sufferers	4	1	30%	–
	No desire for topic-related exchange	2	4	–	20%
Burden related to ET count	Increase in burden with each ET not resulting in pregnancy	2	4	–	20%
Burden related to different treatment phases	Preparatory phase^d^^,^^e^				
	Little	6	2	40%	–
	High	5	2	30%	–
	ET: little	3	–	30%	–
	Waiting time ET—pregnancy test:				
	Little	2	–	20%	–
	High	6	1	50%	–
	Result notification: high	3	–	30%	–
	Lengthy waiting periods between treatment steps: high	3	1	20%	–
	Logistical effort				
	Little	2	–	20%	–
	High	3	1	20%	–
Coping strategies	Verbal exchange	5	2	30%	–
	Couple-centered coping	8	5	30%	–
	Psychological support	2	–	20%	–
	Forums/Instagram/podcasts^b^				
	Experienced positively	4	–	40%	–
	Experienced negatively	4	–	40%	–
Relationship changes	Influence on the bond shared with partner				
	Positive	7	4	30%	–
	Neutral	4	2	20%	–
	Improvement of verbal communication in the relationship	2	–	20%	–
	No increase in conflicts	1	4	–	30%
	Burdensome changes in sexuality			–	
	Yes	3	–	30%	–
	No	2	5	–	30%
Decision-making in fertility treatment	Leaving the decision to their partner	–	3	–	30%
Confounding factors	Personality features^f^ with positive impact on RIF experience	1	4	–	30%
	No previous life events with impact on RIF experience	2	0	20%	–
Use of complementary treatment options	Yes^g^	3	0	30%	–
Consideration of alternative options	Yes^h^	3	5	–	20%
New aspects spontaneously reported	Expectation regarding treatment				
	Was rather naïve/optimistic	6	4	20%	–
	Was rather pessimistic	2	–	20%	–
	Strong emotions toward lost embryo	2	0	20%	–
	Concerns regarding the clinic's financial motivation	0	2	–	20%
	Relativization of negative feelings after successful ET^i^	1	3	–	50%

a Describing rapid and frequent fluctuation in experienced emotional states, moving between both positive and negative valences.

b Meaning negative self-evaluating predictive thoughts.

c Describing the perception of a lack of control and a pessimistic outlook regarding future change.

d Two women had positive and negative views and thus appear in both categories.

e One woman had both positive and negative views and thus appears in both categories.

f Stress resistance and adaptability.

g Traditional Chinese medicine (TCM), acupuncture.

h Egg or sperm donation, adoption, change of fertility clinic.

i At the time of the interview, four couples had achieved a pregnancy after RIF.

### Gender differences in reaction and experiences

Men and women experienced and expressed grief differently (Table 3). Women tended to articulate more elaborately using metaphorical phrasing, and their grief was often accompanied by crying. The emotionally charged loss of an embryo was only reported by women, often described as a valid miscarriage even without evidence of any pregnancy. In contrast, men used more brief descriptions lacking deeper emotional nuances. Feelings of guilt were frequent among women attributing the lack of success directly to themselves, often leading to self-doubt. They also reflected on existential questions, wondering why they had to face these challenges and if they were pursuing something not meant to be, for example, with the following wording.

**Table 3:** Differences between men and women in relation to repeated implantation failure (RIF).

Subcategory	Total, *n* = 20	Women, *n* = 10	Men, *n* = 10
Women's physical experience	10	3	7
Men's greater optimism	5	3	2
Women's greater need for topic-related interpersonal exchange	4	3	1
Gender-specific^a^ burden management	4	4	0
Women's greater wish to have children	4	1	3
Women's more thorough treatment-related research process	4	1	3
Men's tendency to discontinue therapy earlier and undergo fewer ETs	3	2	1

a Men with a more balanced nature, avoidance, and situational awareness gradually over time.

It's like this black hole you fall into after each failed attempt, or also after a miscarriage. For me, it was almost equivalent—whether you lose the pregnancy after one or two weeks or whether you get a negative test. It didn't really make much of a difference. And then you’re in free fall and start doubting yourself intensely. At least for me, that's how it was—at some point, I developed quite a lot of anger toward myself. (Woman)

Women highlighted the strengthened bond with their partners, despite the negative impact on their sexuality and overall quality of life (QOL). The perception of social pressure, meaning the expectation to conform to societal norms, was more of an issue for women than for men. In contrast to men, women found the waiting period after the embryo transfers (ETs) to be the most stressful feeling such as a helpless passive observer, accompanied by uncertainty, tension, and heightened body awareness.

Regarding emotional distress resulting from physical aspects, some women found it highly burdensome, while others tolerated hormone injections, valuing the active involvement. Men linked their increasing burden directly to unsuccessful ETs, equating each failure with diminishing success rates. Women preferred proceeding with treatment despite setbacks, while their partners tended toward discontinuing sooner. Definitive decisions regarding the fertility journey were mostly left to the woman. The use of verbal exchange and couple-centered coping, such as having conversations and working through them together, was more common among women. Men further considered their own individual personality traits helpful, such as stress resistance and adaptability. Using online platforms or psychological support for coping was brought up by women only.

### Gender similarities in reactions and experiences

Men and women experienced helplessness and were disappointed due to their later considered overly optimistic and naïve treatment outcome expectations not being met. Couples struggled with the realization that the outcome was beyond their control and success was not guaranteed. An unclear reason for failed pregnancies intensified uncertainty and deepened a sense of powerlessness.

What is difficult for us—both my husband and me—is that there are no definite answers. You don't know, ‘This is what caused it not to work’ […] It's really like a black box. (Woman)

The topic's sensitive nature led the couple to navigate conversations carefully. They wanted people to refrain from direct inquiries unless sure of the couple's comfort, avoiding indiscretion. Despite good intentions, many outsiders failed to grasp the potential impact of such insensitive questions. Relying on a close circle of friends and family provided valuable support through understanding, attentive listening, and empathy, offering a safe space to vent and express frustration. For couples, the growing treatment duration and burden correlated, but they gradually adjusted to the fertility journey's highs and lows. While initial setbacks hit hardest, they learned to anticipate negative news, integrating it into their attempts to build their family.

[…] Well, I’d say it's really like a rollercoaster. You know, there are all the ups and downs—you’ve gone through them before—and that's really what it is. I’d say we’re slowly starting to come back up now.

(Moderator: So recovering from the last embryo transfer)

Yes, exactly. And, um… So far, every time we started, we were optimistic. We tried to be as positive as possible (laughs), though we always had a little fear that something unexpected might happen. But, um, I think it will turn out fine. (Woman)

Coping strategies such as distraction emerged as effective, allowing individuals to shift their focus and avoid spiraling into negative ruminations. They redirected their energies toward other aspects of life and drew on resources such as personal relationships and health, sports, or humor to alleviate stress. Past life experiences had positive effects on dealing with RIF and shaping coping mechanisms.

[…] I think what helped me most, um… was that a few years ago, I was in psychotherapy for, I’d say, at least two years, maybe even a bit longer, for a mild depression. And I learned a lot about myself, about my emotions, and how to deal with them. I think these tools were extremely helpful to me during that time […]. (Woman)

I think I’m relatively… You can give me quite a lot, and I can handle quite a bit of stress. And I can deal with… all the disappointments, for example, when a big deal doesn't work out. […] and that has certainly helped a bit, in that I’m able to handle the pressure like I have at work a little better, um… yeah, just manage it more effectively. (Man)

Despite complex decision-making and discussions, couples mostly reached a consensus that enhanced relationship harmony (Supplementary Table S1).

### Subjective differences within a couple

Mostly, men cited women's physical experience as a key gender difference. Some women named this aspect as a potential reason why their male partners sometimes struggled to express empathy or felt somewhat like bystanders. Men appeared to focus more often on the positive aspects blocking out the negatives. Women perceived this greater optimism as an advantage, as it helped lift their spirits. Gender differences in burden management were the most frequently noted by women. They reported that their partners did not seek as many conversations or connect with others through social media. Some noted that their male partners increasingly turned to avoidance behavior. Women tended to conduct more research about the treatment process, with men taking a more passive role also regarding communication with health care professionals. Two women indicated that their partners had a relatively weak desire for children. Some men expressed contentment with not having children and feared that the conception process would dominate their lives. Furthermore, there was a tendency among men to discontinue fertility treatment earlier. One man occasionally felt uncertain and left behind during the treatment process, due to his wife's more frequent medical consultations.

### Quantitative interview aspects

**Figure 1:**
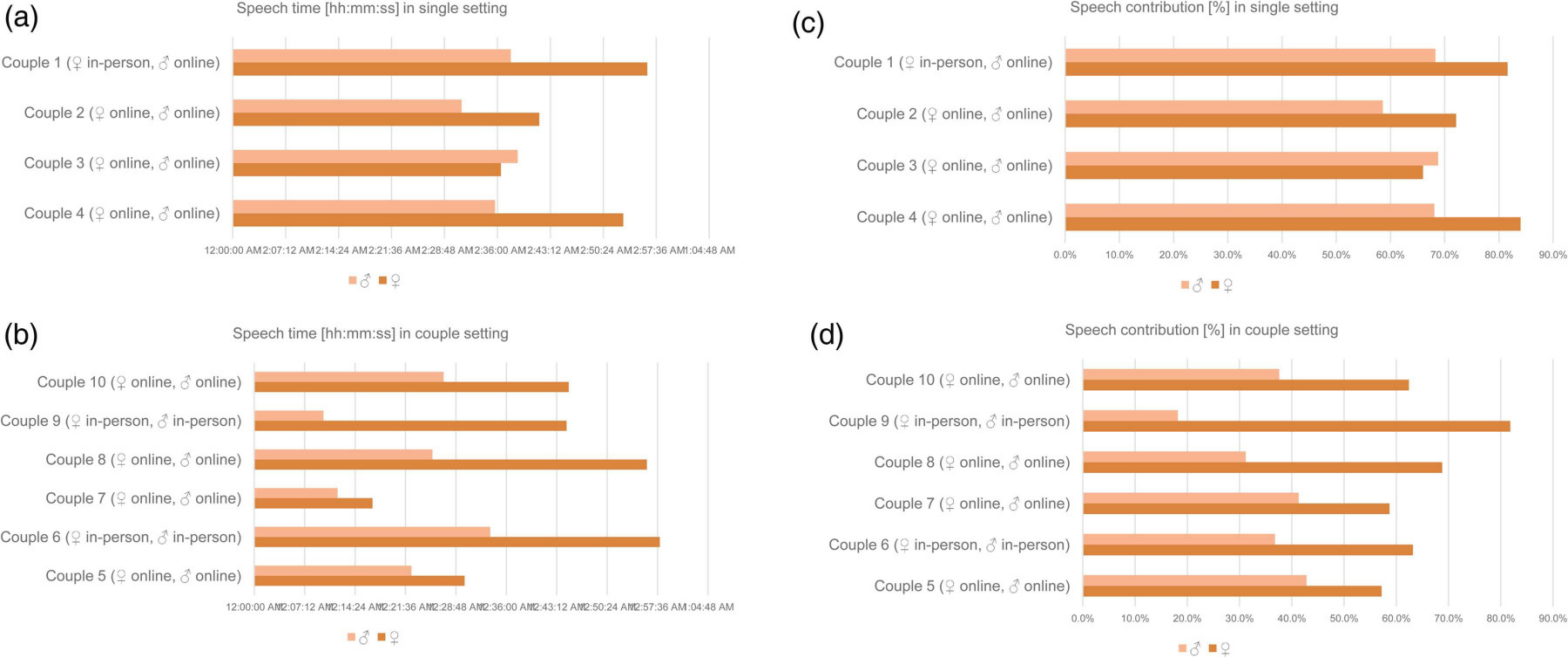
**(a)** Single setting—speech time of four couples separated by sex. **(b)** Couple setting—speech time of six couples separated by sex. **(c)** Single setting—speech contribution of four couples separated by sex. **(d)** Couple setting—speech contribution of six couples separated by sex.

The average in-person speech duration (*n* = 5) was 40:30 ± 19:43 min, compared with 33:58 ± 12:21 min in online interviews (*n* = 15). The qualitative content analysis identified 15 key topics, 121 subcategories, and 581 mentions (57.8% from women). Women contributed 43:49 ± 13:12 min of speaking time compared with 27:23 ± 10:11 min in men. This gender gap persists when differentiated by conversation setting (Table 4; Figures 1a–d). Women provided a similar to substantially increased speech contribution compared with their partners in both the single and couple interview constellations, particularly in joint interviews (Table 5). In male-only interviews, the moderator's speech duration (+27.5%) and contribution (+41.1%) exceeded those in female-only interviews (Supplementary Tables S2a,b). Men contributed fewer speech segments compared with their female partner (−25.3%) on average when interviewed as a couple but more when interviewed separately (+11.3%) (Supplementary Tables S3a,b). Men's average duration of each speech segment was shorter regardless of the interview setting (couple setting, −27.7%; single setting, −34.3%) (Supplementary Table S4). Both men and women showed an increase in their speech segment duration when interviewed individually (Table 4). See Supplementary Tables S2–S4 and Supplementary Figures 1a, b for differences in individual couples.

**Table 4:** Absolute speech time, speech segment count, and duration separated by gender and conversation setting.

	Couple setting		Single setting	
	Absolute speech time			
	♀ (*n* = 6)	♂ (*n* = 6)	♀ (*n* = 4)	♂ (*n* = 4)
Mean ± SD (mm:ss)	41:45 ± 15:45	21:45 ± 09:12	46:55 ± 09:24	35:51 ± 03:24
Range (mm:ss)	16:54–57:55	09:54–33:43	36:28–56:23	31:07–38:45
	Speech segment count			
Mean number ± SD	85.3 ± 34.1	63.7 ± 34.5	62.0 ± 20.4	69.0 ± 15.6
Number range	36–135	31–127	39–86	47–82
	Speech segment duration			
Mean ± SD (s)	30.3 ± 9.0	21.9 ± 9.4	49.9 ± 22.2	32.8 ± 9.6

**Table 5:** Speech contributions separated by gender and interview setting at a couple.

Single setting	Speech contribution (%)		
	♀	♂	
Couple 1	81.6 (in person)	68.3 (online)	
Couple 2	72.1 (online)	58.6 (online)	
Couple 3	66.0 (online)	68.8 (online)	
Couple 4	84.0 (online)	68.1 (online)	
Couple setting	Speech contribution (%)		
	♀	♂	
Couple 5	57.2	42.8	(online)
Couple 6	63.2	36.8	(in person)
Couple 7	58.7	41.3	(online)
Couple 8	68.8	31.2	(online)
Couple 9	81.8	18.2	(in person)
Couple 10	62.4	37.6	(online)

### Couple dynamics

Spontaneous reporting of key topics was analyzed on a couple level, to determine in which subcategory the number of concordances (both partners vocalized opinions on a specific key topic) and discordances (only one partner vocalized an opinion on a specific key topic) was most obvious. There was no distinction made regarding the quality or content of the statement, focusing solely on the process of addressing a topic. Across all subcategories, there were fewer concordances (*n* = 122) than discordances (*n* = 272). Men were overall less likely to address a key topic, especially regarding the use of online platforms and the experience of guilt. The subcategories *growing burden with increasing duration of treatment* and *distraction and shift of focus as a coping strategy* demonstrated the greatest concordance. Controlling for the conversation setting, there were almost twice as many concordances when the couples conducted the interview together (*n* = 81), compared with a single setting (*n* = 45).

## Discussion

To provide a reliable basis for the amelioration of current support models, this interview study aimed to determine whether and how women's and men's responses to RIF differ.

On average, women contributed a verbal majority in the interview. Men's tendency to address a smaller range of core topics was less evident in a couple setting. Guilt, grief, and struggling with fate were core emotional reactions predominantly seen in women and, if expressed by men, were less intensive and lacking metaphorical description. Male reactions were dominated by disappointment, which emerged, alongside helplessness, as a core gender similarity. Men primarily cited women's physical experience as a key gender difference while gender differences in coping were mentioned the most by women. Men, having the more passive role, perceived fertility treatment as less burdensome with a smaller impact on QOL and sexuality. Waiting periods after ET were especially burdensome for the women. With increased treatment duration, a certain level of mental adjustment to failure was reported in men and women with the former showing a tendency to discontinue treatment sooner. Men found their traits and past life experiences helpful in coping with RIF. The most evident shared strategy is a concept of distraction and shifting focus in life. Similar to women, men, although feeling less need for social support, preferred a small circle of confidants. The following listing gives a short overview of gender similarities and differences:

Similarities between reactions to RIF in men and women:

- Feelings of helplessness and disappointment
- Overly optimistic treatment outcome expectations
- Careful navigation of conversations with external parties
- Emotional adaptation to failure
- Distractions experienced as helpful coping strategies

Differences between reactions to RIF in men and women:

- Negative impact on QOL and sexuality despite a positive impact on partner bond
- Emotional expression and narrative style
- Feelings of guilt, struggling with fate
- Perception of social pressure
- Decision-making regarding the timeline of the fertility journey
- Choice of coping strategies: verbal exchange, online platforms, professional psychological help

### Differing reactions to RIF between men and women

Core reactions such as grief, struggling with fate, and guilt emerged as gender differences, with women experiencing these more intensely. Given that, in cases of infertility, one might cynically label a “culprit” depending on the failed pregnancy's biological cause, the emerging topic of guilt within partnerships is expected (18–20) and may influence coping with RIF. Women feeling self-blame echoed existing literature (21); however, in our sample, this was irrespective of the underlying cause of infertility. Men exhibiting a substantially lower speech time may suggest that they either struggle to grasp and express their feelings verbally or react less emotionally to RIF. Men reported a smaller number of psychosocial reactions indicating a less burdensome perception and resulting distress, supporting other findings (22, 23). In our study, QOL was assessed based on the extent to which the unfulfilled desire for children occupies participants' lives. As only women expressed their thoughts on this matter, this supports previous findings where men's QOL was not significantly reduced (24). This may indicate that the unfulfilled desire to build a family has a greater impact on women's lives, with this disparity being potential fuel for conflict. Men's most reported gender difference was the physical part of treatment borne by women, emphasizing an externalized, physical focus. The unexpected neutral impact on the self-perception of being a fully valid man or woman in society might be explained by less rigid gender roles and therefore social expectations, in more liberal countries (25). However, women still reported perceived social pressure which may stem from the social stigma of infertility (26, 27). The negative impact on sexuality, predominantly experienced by women, was another more apparent gender difference than described in the literature (21). In line with the concept of marital benefit in fertility treatment (28, 29), no negative impact on the couples' bond was observed. Couple-centered coping as a contributing factor may have counteracted the trend of relationships becoming less stable with an increasing number of unsuccessful treatments (30).

The reported gender gap in information gathering and linked decision-making power could be related to the sample's educational level, as previous research stated that particularly educated women are more likely to do internet research about fertility treatment (31). However, it is also known that men in general tend to seek less online health information (32). Since most treatment phases are focused on the female body, their greater interest is reasonable. Men, often feeling rather excluded and finding their needs insufficiently considered in current care concepts due to the greater focus on women (33), corresponded notably only to a minority of our male participants, which might be a result of the clinic’s effort to integrate partners wherever possible and desired by the couple.

### Differing coping strategies for RIF between men and women

Revealed coping differences in RIF were less pronounced than expected (5, 34, 35). Some patterns, such as women mentioning a broader spectrum of strategies and men seeking less psychological and social support with a smaller need for verbal exchange, were in full agreement with current literature (35–38). Unexpectedly (38), there was no male dominance in distraction as a coping strategy. However, men's tendency toward avoidance was often estimated higher by their partners, indicating an important potential perception gap as an individual's avoidant coping strategy can increase partner stress (39).

### Interview setting and quantitative conversation parameters

Although logistical factors primarily shaped the interview setting, unspoken aspects of introverted or shy personality traits must not be neglected. Even if not explicitly stated, the threshold for disclosed discussion of sensitive topics as such is generally lower in an online setting (40). For our observation of shorter duration of online interviews (40, 41), differing conversation dynamics, intrinsic motivation, and time investment might be the causative factors. Although there are opposite findings (40), a participant taking the time to attend an in-person interview may be more willing to discuss the topic in a lengthy fashion. Men's lower speech contribution found in our study aligns with current literature, stating that women's perspectives are more present in joint interviews (42). This may reflect verbal exchange as a dominant coping strategy for women, who might have engaged more extensively with their thoughts and emotions. In our case, the gender of the interviewer matching the interviewee's may have had an influence on the capacity of empathy and relatability, as the moderator is an essential component in a dyadic interview structure (43).

### Couple dynamics influencing communication

Infertility affects a couple as a dyad, implying significant interpersonal influence, blurring the distinction between couple and gender differences in our study. Dyadic influence is directly present when the interview is conducted as a couple (43), reflecting the higher number of concordances within our couples. However, concordance was only assessed by analyzing whether both partners spontaneously vocalized a topic, including any form of affirming verbal cues, indicating a relevant personal interest. It remains unclear if these verbal cues represent the likelihood of spontaneous verbalization also in an individual setting. Most discordances had to be attributed to the reduced likelihood of men addressing a topic. Dyadic interview structure may reduce the influence of socially desired stances on a partner's answer (43), although this does not necessarily mean that given statements represent an individual partner's unbiased opinion, as the desire to avoid disagreement and prevent conflict triggers might have an impact. Social desirability and conflict avoidance might have resulted in more congruent answers. By leaving the choice of the setting to the study participants, we aimed to open opportunities to integrate any desired input into the study findings. However, the dyadic influence in conversations with both partners should be taken into consideration in clinical settings.

In addition to the verbal cues we analyzed in the present studies, emotional and psychosocial reactions might also have been translated into specific coping and supporting actions, which would also need to be investigated to get the full picture.

### Strengths and limitations

Aside few other studies (44, 45), it is novel to see men's perspective displayed to this extent. The sample size reaching saturation is found to be within the typical range for this study type (46, 47). The interviewer was female, which might have influenced responses as study participants might expect such an interviewer to be more supportive of the women and follow estimated social desirability in their answers. In addition, a female interviewer may be seen as less able to understand male reactions, which might have hampered disclosure, especially of very personal and critical reactions. With the content analysis's coding process carried out by a single person, intercoder reliability is not provided, which might have resulted in overlooking as well as overestimating responses. Due to ongoing adjustments of the interview guide, there is an inhomogeneous set of questions. However, being already very comprehensive, topics intrinsically important to participants were addressed upon personal motivation. Data were self-reported, i.e., definitions of specific factors and experiences may vary. Difficulties in self-assessment, mood, and well-being of the respondent at the time of the interview, and individual honesty might have influenced our results. The interview settings were not balanced and led to a heterogenous data collection. Although the predefined interview guide guaranteed that all key topics were covered, the heterogeneity of interview models might have resulted in a different degree of differentiation as well as openness. While all the study participants were Caucasian and mostly highly educated, age and duration of infertility differed, so that background conditions were inhomogeneous. The size of the study group did not allow for any subanalysis to evaluate the association between background factors and findings. The significant range in time between the last ET and the interview could contribute to recall bias, i.e., especially negative emotions might be remembered more strongly when the disappointment of another negative pregnancy test was experienced only recently. However, against our expectations of changes with each ET not resulting in a pregnancy, most study participants estimated their overall reaction as more relevant than specific reactions to each ET. Due to Switzerland's self-pay system for *in vitro* fertilization (IVF)/intracytoplasmic sperm injection (ICSI) treatments, the selection of a homogenous study sample regarding wealth and cultural background was expected; however, this makes our results not universally applicable to other cultures, for example, non-Western cultures. Although there are individual differences in Switzerland and nearby nationalities, women and men are perceived as relatively equivalent, so that aiming for a family is considered a project of both partners, which strongly differs from countries where women are seen as the main person responsible for a successful pregnancy. Such cultural differences likely influence feelings of guilt and social pressure (47), so that further studies including participants with different cultural backgrounds are needed. The high male participation rate of 77% likely reflects self-selection bias, as participating male partners may be more understanding, supportive, empathetic, and motivated. As a consequence, their responses might represent a more favorable attitude toward their partner and participation in fertility treatment, while reactions of less interested and caring partners are missing.

### Clinical implications

Based on the interview findings, the Zurich University Hospital's Department of Reproductive Endocrinology is preparing a longitudinal project on broader collectives to optimize the clinic's support systems and care concepts. Knowledge emerging from this analysis was integrated into the fertility treatment process. Specifically, these findings served to improve counseling sessions, redefining key topics and integrating gender-specific coping interventions.

**Figure 2:**
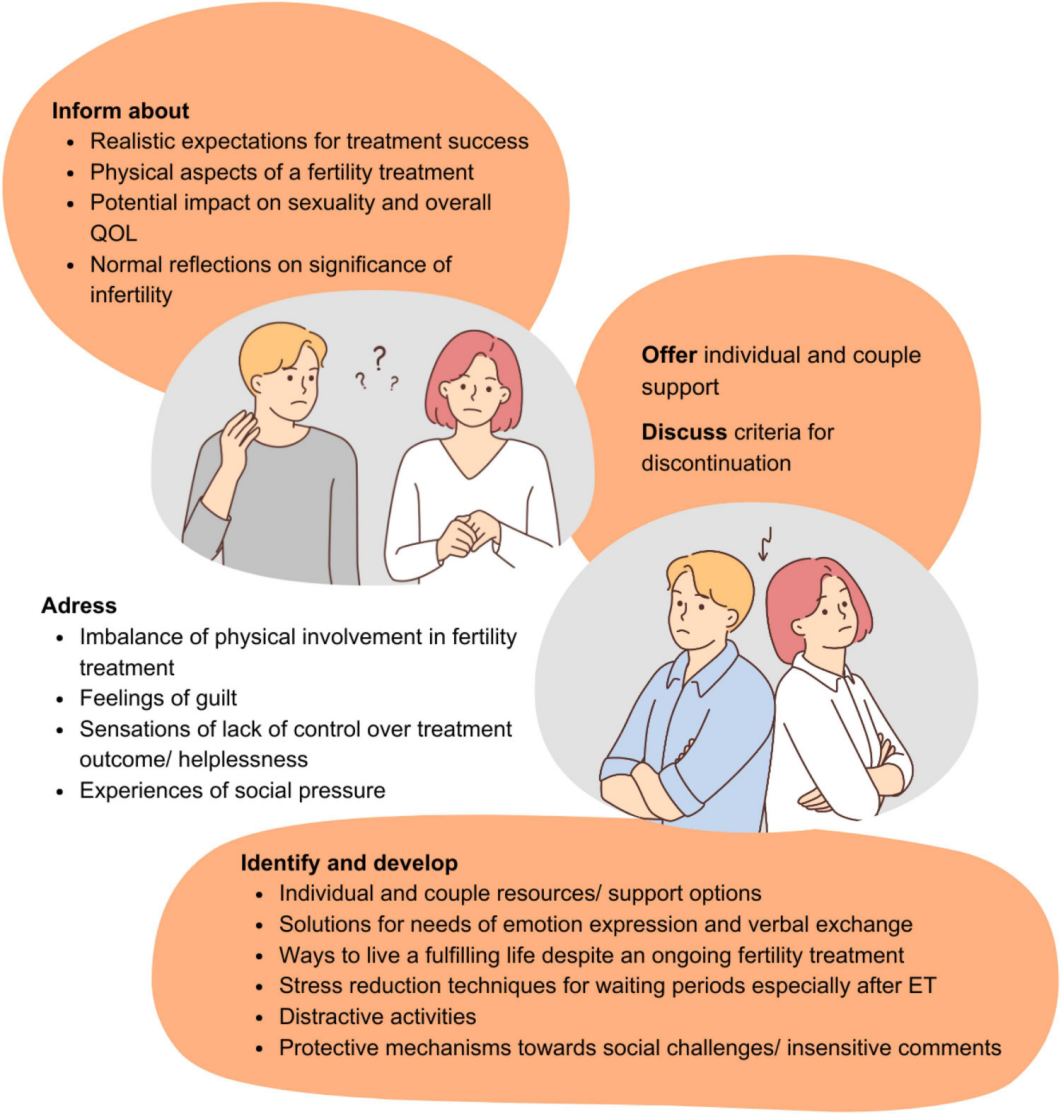
General and gender-specific aspects of a counselling model for couples experiencing RIF. Pictograms created with Canva (http://www.canva.com).

Based on our findings, a counseling model for couples experiencing RIF should consider the general and gender-specific aspects visualized in Figure 2. Such counselling should be offered either directly by fertility specialists or by other trained healthcare professionals irrespective of the sociocultural background of the couple experiencing RIF.

### Future studies

Future studies should include women where the partner does not want to participate, study participants with different cultural backgrounds, and those receiving other ART modalities, for example, heterologous treatments or surrogacy, as they are associated with further emotional and psychosocial adaptations. Ideally, they should go beyond the treatment phase and also evaluate long-term relationships and developmental outcomes in a multidisciplinary, longitudinal design to better understand evolving psychosocial impact. To increase reliability, they should use a two-coder approach with intercoder reliability checks at defined points of the investigation.

## Conclusion

The qualitative analysis of emotional and psychosocial effects of RIF showed its complexity, underlining the importance of considering gender aspects which underly a dyadic influence in couples. Men generally reported less diverse psychosocial reactions and a narrower, slightly differing spectrum of coping strategies than women. A key finding revolves around the issue of guilt being an important aspect in partnership dynamics. Results arising from this study should serve as a basis for process optimization, including inclusive and gender-specific support offers to better accompany affected couples as two individuals and improve their psychological well-being during fertility treatment.

## Data Availability

The raw data supporting the conclusions of this article will be made available by the authors, upon reasonable request.
