# Supplementary material for: Gender differences in dealing with recurrent implantation failure after fertility treatments: a foundation for adequate support models
Source: Front Reprod Health. 2025 Nov 17;7:1698380. doi: 10.3389/frph.2025.1698380 (PMC12665732; doi:10.3389/frph.2025.1698380)
Supplement: Supplementary file 1 [file Table1.docx]

**Supplementary Tables**

**Supplemental table 1: Similarities – Effects of repeated implantation failure mentioned by ≥40% of the participants and in which men and women differ by ≤10%.**

| **Experiences** | **Specification** | **Women**  **n = 10** | **Men**  **n = 10** | ♀ **> ♂** | **♂ > ♀** |
| --- | --- | --- | --- | --- | --- |
| **Emotional and psychological well-being** | Disappointment and Disillusionment | 6 | 7 | - | 10% |
|  | Feelings of helplessness | 4 | 3 | 10% | - |
| **Social pressure** | Questions about fertility topic perceived as crossing boundaries/insensitive/ intrusive | 4 | 3 | 10% | - |
| **Social support** | Wish for topic-related exchange with trusted individuals | 5 | 4 | 10% | - |
| **Treatment duration dependency** | Growing burden with increasing duration of treatment | 9 | 9 | - | - |
|  | Initiation of adaptation process with duration of treatment | 4 | 3 | 10% | - |
| **Coping strategies** | Distraction and shift of focus | 9 | 9 | - | - |
|  | Use of resources^1^ | 4 | 5 | - | 10% |
| **Relationship changes** | Challenges arising experienced as a burden | 4 | 4 | - | - |
| **Decision-making in fertility treatment** | Unified opinion shared by the couple | 5 | 4 | 10% | - |
| **Confounding factors** | Previous life events with positive impact on RIF experience^2^ | 5 | 4 | 10% | - |

*Note. ^1^I.e. personal relationships and health, sports, humor. ^2^I.e. personal illness, loss of a loved one, work-related stress and other unspecified life challenges.*

**Supplemental table 2a: Overview of speech contributions at a couple level separated by interview setting with consideration of moderator.**

| **Single setting** |  |  |
| --- | --- | --- |
|  | **Speech contribution ♀ [%]** | **Speech contribution ♂ [%]** |
| Couple 1 | 81.6 *(in person)*  Moderator: 18.4 | 68.3 *(online)*  Moderator: 31.7 |
| Couple 2 | 71.5 *(online)*  Moderator: 28.5 | 58.6 *(online)*  Moderator: 41.4 |
| Couple 3 | 66.0 *(online)*  Moderator: 34.0 | **68.8** *(online)*  Moderator: 31.2 |
| Couple 4 | 84.0 *(online)*  Moderator: 16 | 68.2 *(online)*  Moderator: 31.8 |

| **Couple setting** |  | |  | |
| --- | --- | --- | --- | --- |
|  | **Speech contribution ♀ [%]** | **Speech contribution ♂ [%]** | | **Speech contribution moderator [%]** |
| Couple 5  (online) | 41.8 | 31.3 | | 26.9 |
| Couple 6  (in person) | 54.2 | 31.5 | | 14.3 |
| Couple 7  (online) | 39.3 | 27.7 | | 33.0 |
| Couple 8  (online) | 57.9 | 26.3 | | 15.8 |
| Couple 9  (in person) | 52.3 | 11.6 | | 36.1 |
| Couple 10  (online) | 49.5 | 29.8 | | 20.7 |

*Note.* *If the male outperforms the female, it is emphasized in bold.*

**Supplemental table 2b: Mean speech contributions separated by interview setting with consideration of moderator.**

|  | **Couple setting** | | **Single setting** | |
| --- | --- | --- | --- | --- |
|  | **Distribution of speech contributions** | | |  |
| **Mean ± SD [%]** | ♀ (n = 6): | 49.2 ± 7.3 | ♀ (n = 4):  Moderator: | 75.8 ± 8.5  24.2 ± 8.4 |
|  | ♂ (n = 6): | 26.4 ± 7.5 | ♂ (n = 4):  Moderator: | 66.0 ± 4.9  34.0 ± 4.9 |
|  | Moderator: | 24.5 ± 9.0 |  |  |

| **Couple setting** | **Speech contribution [%]** | | |
| --- | --- | --- | --- |
|  | **♀** | **♂** |  |
| Couple 5 | 57.2 | 42.8 | *(online)* |
| Couple 6 | 63.2 | 36.8 | *(in person)* |
| Couple 7 | 58.7 | 41.3 | *(online)* |
| Couple 8 | 68.8 | 31.2 | *(online)* |
| Couple 9 | 81.8 | 18.2 | *(in person)* |
| Couple 10 | 62.4 | 37.6 | *(online)* |

**Supplemental table 3a: Absolute speech time, speech segment count and duration separated by gender and conversation setting.**

|  | **Couple setting** |  | **Single setting** |  |
| --- | --- | --- | --- | --- |
|  | **Absolute speech time** | | | |
|  | ♀ (n = 6) | ♂ (n = 6) | ♀ (n = 4) | ♂ (n = 4) |
| **Mean ± SD [mm:ss]** | 41:45 ± 15:45 | 21:45 ± 09:12 | 46:55 ± 09:24 | 35:51 ± 03:24 |
| **Range [mm:ss]** | 16:54 - 57:55 | 09:54 - 33:43 | 36:28 - 56:23 | 31:07 - 38:45 |
|  | **Speech segment count** | | | |
| **Mean number** **± SD** | 85.3 ± 34.1 | 63.7 ± 34.5 | 62.0 ± 20.4 | 69.0 ± 15.6 |
| **Range** | 36 - 135 | 31 - 127 | 39 - 86 | 47 - 82 |
|  | **Speech segment duration** | | | |
| **Mean ± SD [ss]** | 30.3 ± 9.0 | 21.9 ± 9.4 | 49.9 ± 22.2 | 32.8 ± 9.6 |

**Suppl. Table 3b: Speech contributions separated by gender and interview setting at a couple.**

| **Single setting** | **Speech contribution [%]** | |
| --- | --- | --- |
|  | **♀** | **♂** |
| Couple 1 | 59.8 *(in person)* | 40.2 *(online)* |
| Couple 2 | 57.3 *(online)* | 42.7 *(online)* |
| Couple 3 | 48.5 *(online)* | **51.5** *(online)* |
| Couple 4 | 59.8 *(online)* | 40.2 *(online)* |

**Supplemental table 4: Overview of speech segment count and average duration separated by interview settings.**

| **Single setting** |  |  |  |  |
| --- | --- | --- | --- | --- |
|  | **Speech segments ♀ [n]** | **Speech segments ♂ [n]** | **Average duration of speech segment ♀ [mm:ss]** | **Average duration of speech segment ♂ [mm:ss]** |
| Couple 1 | 86 *(in person)* | 78 *(online)* | 00:39 *(in person)* | 00:29 *(online)* |
| Couple 2 | 53 *(online)* | **82** *(online)* | 00:47 *(online)* | 00:23 *(online)* |
| Couple 3 | 70 *(online)* | 69 *(online)* | 00:31 *(online)* | **00:34** *(online)* |
| Couple 4 | 39 *(online)* | **47** *(online)* | 01:22 *(online)* | 00:46 *(online)* |

| **Couple setting** |  |  |  |  |
| --- | --- | --- | --- | --- |
| Couple 5  (online) | 73 | 31 | 00:25 | **00:43** |
| Couple 6  (in person) | 135 | 127 | 00:26 | 00:16 |
| Couple 7  (online) | 36 | **39** | 00:28 | 00:18 |
| Couple 8  (online) | 70 | 50 | 00:48 | 00:31 |
| Couple 9  (in person) | 108 | 63 | 00:25 | 00:09 |
| Couple 10  (online) | 90 | 72 | 00:30 | 00:23 |

*Note. If the male outperforms the female, it is emphasized in bold.*

**Supplementary Figures**

**Supplemental figure 1a: Single setting – speech time of the ten couples separated by conversation setting.**

**Supplemental figure 1b: Couple setting – speech time of the ten couples separated by conversation setting.**
